# Increased levels of HMGB1 and pro-inflammatory cytokines in children with febrile seizures

**DOI:** 10.1186/1742-2094-8-135

**Published:** 2011-10-11

**Authors:** Jieun Choi, Hyun Jin Min, Jeon-Soo Shin

**Affiliations:** 1 Department of Pediatrics, Seoul National University Boramae Hospital, Seoul National University, College of Medicine, Seoul, Korea; 2 Department of Microbiology, Yonsei University College of Medicine, Seoul, Korea; 3 Severance Biomedical Science Institute and Institute for Immunology and Immunological Diseases, Yonsei University College of Medicine, Seoul, Korea

## Abstract

**Objective:**

Febrile seizures are the most common form of childhood seizures. Fever is induced by pro-inflammatory cytokines during infection, and pro-inflammatory cytokines may trigger the development of febrile seizures. In order to determine whether active inflammation, including high mobility group box-1 (HMGB1) and pro-inflammatory cytokines, occurs in children with febrile seizures or epilepsy, we analyzed cytokine profiles of patients with febrile seizures or epilepsy.

**Methods:**

Forty-one febrile seizure patients who visited the emergency department of Seoul National University Boramae Hospital from June 2008 to May 2009 were included in this study. Blood was obtained from the febrile seizure child patients within 30 minutes of the time of the seizure; subsequently, serum cytokine assays were performed. Control samples were collected from children with febrile illness without convulsion (N = 41) and similarly analyzed. Serum samples from afebrile status epilepticus attacks in intractable epilepsy children (N = 12), afebrile seizure attacks in generalized epilepsy with febrile seizure plus (GEFSP) children (N = 6), and afebrile non-epileptic controls (N = 7) were also analyzed.

**Results:**

Serum HMGB1 and IL-1β levels were significantly higher in febrile seizure patients than in fever only controls (*p *< 0.05). Serum IL-6 levels were significantly higher in typical febrile seizures than in fever only controls (*p *< 0.05). Serum IL-1β levels were significantly higher in status epilepticus attacks in intractable epilepsy patients than in fever only controls (*p *< 0.05). Serum levels of IL-1β were significantly correlated with levels of HMGB1, IL-6, and TNF-α (*p *< 0.05).

**Conclusions:**

HMGB1 and pro-inflammatory cytokines were significantly higher in febrile seizure children. Although it is not possible to infer causality from descriptive human studies, our data suggest that HMGB1 and the cytokine network may contribute to the generation of febrile seizures in children. There may be a potential role for anti-inflammatory therapy targeting cytokines and HMGB1 in preventing or limiting febrile seizures or subsequent epileptogenesis in the vulnerable, developing nervous system of children.

## Background

Febrile seizures are the most common form of childhood seizures, occurring in 2%-5% of children younger than 6 years old [1]. Febrile seizures are defined as seizures that occur during a febrile state and without an obvious central nervous system infection. Fever is induced by pro-inflammatory cytokines such as interleukin (IL)-1β, IL-6, and tumor necrosis factor (TNF)-α during infections. The fever threshold temperature for febrile seizures varies among individuals, as well as by age and maturation [2]. Genetic susceptibility to inflammation may influence the fever threshold temperature for febrile seizures, and 17-30% of febrile seizure patients have a family history of febrile seizures [2]. IL-1β biallelic polymorphism in the promoter region at the -511 position is significantly higher in febrile seizure patients than in fever only children, and this polymorphism results in an increase in IL-1β production [3,4]. However, others have failed to demonstrate a significant association between IL-1β (-511) and febrile seizures [5,6]. The association of IL-1β gene polymorphism and susceptibility to febrile seizures is still controversial. Increased levels of IL-6, and IL-1-receptor antagonist/IL-1β ratio have been reported in the plasma of febrile seizure patients [3]. Viruses as causative agents of febrile seizures have been demonstrated in several reports. Neurotropic viruses, such as herpes and influenza A, are commonly associated with febrile seizures [7,8].

Pro-inflammatory cytokines may trigger febrile seizures. In experimental animals, intraventricular injection of IL-1β reduces the seizure threshold in 14-day old mice subjected to hyperthermia, while IL-1β knock-out mice had an increased seizure threshold [9]. IL-1β increases glutamatergic neurotransmission and lowers the peak magnitude of GABA-mediated currents [10], supporting the role of pro-inflammatory cytokine contribution to the generation of fever-induced seizures [9]. Also, IL-1β prolongs the duration of electroencephalographic seizure [11].

High mobility group box-1 (HMGB1) has been shown to be a key mediator of inflammatory diseases. HMGB1 is a nuclear protein that triggers inflammation, binds to lipopolysaccharides (LPS) and IL-1, and initiates and synergizes with a Toll-like receptor (TLR) 4-mediated pro-inflammatory response [12]. After pro-inflammatory stimulation, such as that by LPS, TNF-α, IL-1, IL-6 and IL-8, HMGB1 is actively released from activated monocytes and macrophages. Regulation of HMGB1 secretion is important for control of HMGB1-mediated inflammation and is dependent on various processes such as phosphorylation by calcium-dependent protein kinase C [13], as well as acetylation and methylation [14]. In a recent study, HMGB1 and TLR4 were involved in the generation and recurrence of seizures in experimental animals [15,16].

Cytokine analyses in our previous study showed that pro-inflammatory cytokine levels, including IL-1β, IL-8, IL-12p70, and macrophage inflammatory protein (MIP)-1β, were significantly high in the epileptogenic cortex of intractable epilepsy children [17]. In addition, levels of IL-6 and MCP-1 were significantly high in patients with a family history of epilepsy. Active neuroinflammation, such as a marked activation of microglia and astrocytes as well as marked cellular injury, were also observed in epileptogenic brain tissue, supporting the suggestion that neuroinflammation may contribute to epileptogenesis in the developing brain.

In order to determine whether active inflammation, including HMGB1 and pro-inflammatory cytokines, occurs in children with febrile seizures and pediatric epilepsy, we analyzed cytokine profiles in the serum of child patients with febrile seizures or epilepsy and assessed the correlation between cytokine levels and febrile seizures.

## Materials and methods

### Patient information

Forty-one febrile seizure patients who visited to emergency department of Seoul National University Boramae Hospital from June 2008 to May 2009 were included in this study (Table 1). Blood was obtained from patients within 30 minutes of the time of seizure, and serum was immediately separated and frozen for subsequent cytokine assay. Patient inclusion criteria were age between 6 months and 6 years, body temperature ≥38.5°C, C-reactive protein (CRP) ≤2.0, and presented no other identifiable cause of the seizure. Clinical data for familial febrile seizure history, earlier febrile seizure attacks, as well as duration and semiology of febrile seizures were obtained from the patients' parents. Family history was regarded as positive when febrile seizures occurred in first-degree relatives. Laboratory findings, including complete blood counts (CBC), blood chemistry, and CRP, were checked at the time of seizure. CRP levels higher than 2.0 were excluded due to presumptive presence of bacterial infection. Febrile seizure patients were classified into two types: typical type for whom febrile seizures persist for < 15 minutes, are generalized tonic-clonic, and only occur once within 24 hours; and atypical types for whom seizures persist for > 15 minutes, or are partial seizures, or recur within 24 hours of the initial attack. Control samples were collected from children with febrile illness, but without convulsion (N = 41). Control groups were matched for age and temperature criteria and had no convulsions during the febrile illness and no known history of previous febrile seizures. Control blood serum was collected and frozen as above. In addition, blood serum was collected and frozen from afebrile status epilepticus attacks in intractable epilepsy children (N = 12), afebrile seizure attacks in GEFSP children (N = 6), and afebrile non-epileptic controls (N = 7) for cytokine assay in order to subtract fever effects from the cytokine levels. The study was approved by the Institutional Review Board at the Seoul National University Boramae Medical Center (20080918/06-2008-74/76). Informed consent was obtained from each child's parents.

**Table 1:** Clinical findings of febrile seizure, epilepsy, and control children

	Fever only control (N = 41)	Febrile seizure (N = 41)	Afebrile control (N = 7)	Afebrile seizure (N = 6)	Afebrile SE (N = 12)
Age (year)	3.1	2.1	7.9	7.6	6.3
Male/Female	24/17	29/12	3/4	4/2	8/4
BT at admission(°C)	38.4	39.0	36.6	37.0	36.5
WBC count (per mm^3^)	11,719	12,068	7,540	8,716	9,560
CRP	0.91	0.95	0.4	0.6	0.8

SE, status epilepticus; BT, body temperature

### Cytokine measurement

Levels of pro-inflammatory cytokines including HMGB1, IL-1β, IL-6, interferon (IFN)-β, TNF-α, and anti-inflammatory cytokine IL-10 were measured using commercially available, enzyme-linked immunosorbent assay (ELISA) kits according to the manufacturer's instructions (for HMGB1, Shino-Test Corp., Tokyo, Japan [17]; for IL-1β, IFN-β, TNF-α, and IL-10, Panomics Inc., Redwood City, CA, USA; for IL-6, R&D Systems, Minneapolis, MN, USA). Samples were analyzed in duplicate and compared with controls. The detection limits were 0.2 ng/mL for HMGB1, 0.27 pg/mL for IL-1β, 0.23 pg/mL for IL-6, 0.21 pg/mL for IFN-γ, 0.49 pg/mL for TNF-α and 0.22 pg/mL for IL-10.

### Statistical Analysis

The χ^2 ^test was used to compare the clinical characteristics between febrile seizure patients and the controls. The Mann-Whitney test was used to compare serum cytokine levels and laboratory findings between controls and febrile seizure patients. The Spearman's rank correlation coefficient was calculated to detect significant correlations between cytokine levels. The Kruskal Wallis test was used to compare cytokine levels among afebrile controls, febrile controls, and four seizure groups (first attack febrile seizure, recurrent attack febrile seizure, afebrile seizure attack in GEFSP, and afebrile status epilepticus attacks in intractable epilepsy patients). GraphPad Prism v. 4.0 (GraphPad Software Inc., San Diego, CA, USA) was used to perform the above tests. Values are expressed as means, and statistical significance of differences was set as *p *< 0.05 for all tests.

## Results

### Patient characteristics

Table 1 summarizes the patient's clinical data. Forty-one febrile seizure patients and 41 control children with febrile illness without convulsion were included in this study. The mean age of febrile seizure patients was 2.1 years. Boys were more prevalent than girls were (respectively, 71% vs. 29%). Eleven (27%) patients had a family history of febrile seizures and fourteen (34%) patients exhibited atypical types of febrile seizures (Table 2). Twenty-eight patients (68%) had their first febrile seizure attack and thirteen patients (32%) had experienced previous febrile seizure attacks. Febrile seizure patients and febrile children without seizures did not significantly differ by sex, age, and laboratory data.

**Table 2:** Subgroups of febrile seizure patients

	Febrile seizure patients (N = 41)	
Family history of FS	Positive	11 (27%)
	Negative	30 (73%)

FS history	First attack	28 (68%)
	Recurrent	13 (32%)

FS type	Typical	27 (66%)
	Atypical	14 (34%)

FS, febrile seizure

### Serum cytokine levels in the febrile seizure patients; increased IL-1β, IL-6, IL-10 and HMGB levels

In febrile seizure patients, serum IL-1β levels were at a 4-fold increase and HMGB1 levels were at a 1.3-fold increase higher than the fever only controls (Table 3, both *p *< 0.05). Serum levels of IL-6 were at a 1.8-fold increase and IL-10 were at a 2.8-fold increase in febrile seizure patients higher than the fever only controls, although statistically not significant (Table 3, *p *= 07 and *p *= 0.05). There were no differences in serum IFN-γ and TNF-α levels between febrile seizure patients and fever only controls (Table 3).

**Table 3:** Comparisons of cytokine levels between fever only control and febrile seizure subgroups

Groups (No.)	IL-1β	IL-6	HMGB1	IFN-γ	TNF-α	IL-10
	(pg/mL)	(pg/mL)	(ng/mL)	(pg/mL)	(pg/mL)	(pg/mL)
**Fever only control **(41)	3.1 ± 0.8†	134.0 ± 22.7	24.8 ± 2.5	84.2 ± 38.6	5.6 ± 2.9	8.3 ± 2.6

**Febrile seizures **(41)	12.0 ± 5.3*	247.1 ± 43.0	32.6 ± 3.0*	73.5 ± 20.9	5.0 ± 1.8	23.6 ± 13.4

Typical FS (27)	12.9 ± 7.6*	260.1 ± 50.4*	30.5 ± 3.8	66.5 ± 21.6	3.0 ± 0.9	28.5 ± 20.3

Atypical FS (14)	10.4 ± 4.9	228.3 ± 81.2	36.6 ± 5.0*	86.9 ± 45.9	9.0 ± 5.0	14.2 ± 3.8

First FS (28)	3.7 ± 0.7	252.7 ± 53.5	35.0 ± 3.9*	74.6 ± 21.3*	1.9 ± 0.6	9.2 ± 2.2

Recurrent FS (13)	30.1 ± 15.7	241.7 ± 73.0	27.3 ± 4.7	70.9 ± 48.8	11.8 ± 5.3	54.6 ± 41.8

FS without FHx (30)	13.0 ± 6.9	241.6 ± 49.3	31.8 ± 3.6	62.8 ± 22.5	5.2 ± 2.4	29. 4 ± 18.2*

FS with FHx (11)	9.4 ± 5.6	270.1 ± 89.2	34.7 ± 5.9	102.6 ± 48.5	4.7 ± 2.1	7.8 ± 2.7

* indicates a significant (*p *< 0.05) difference compared to fever only control (Mann-Whitney test).

FS, febrile seizure; FHx, family history; SE, status epilepticus

†; mean ± standard error of mean

In comparisons of the subgroups of febrile seizure patients with the fever only control, typical febrile seizure patients showed a 4.2-fold increase of IL-1β and a 1.9-fold increase of IL-6 levels higher than the fever only controls (Table 3, both *p *< 0.05). Both atypical febrile seizure and first attack febrile seizure patients showed a 1.5- and a 1.4-fold increase of HMGB1 levels higher than the fever only controls (Table 3, both *p *< 0.05). The IL-1β levels were at an 8.1-fold increase in patients with recurrent febrile seizure attacks than those with first febrile seizure attack, although statistically not significant (Table 3, *p *= 0.27). IL-10 levels showed a 6.6-fold increase in children with recurrent febrile seizure attacks higher than the fever only controls (Table 3, *p *= 0.06). Febrile seizure patients without a family history of febrile seizures showed a 3.5-fold increase of IL-10 levels (Table 3, *p *< 0.05) above the fever only controls and a 3.7-fold increase above those with FS without a family history of febrile seizures (Table 3, *p = *0.31).

### Serum cytokine levels in the afebrile control, febrile control, and afebrile various seizure groups

#### 1. IL-1β

**Figure 1:**
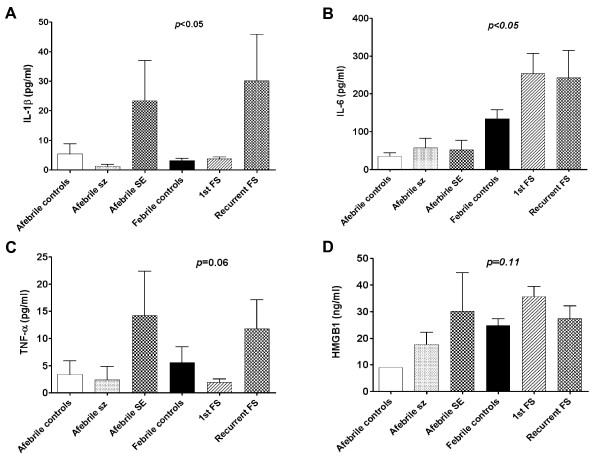
**Serum cytokine levels in different seizure patients**. (A-D) Mean serum cytokine levels of IL-1β (A), IL-6 (B), TNF-α (C), and HMGB1 (D) in afebrile control (N = 7), afebrile seizure (sz) attacks in generalized epilepsy with febrile seizure plus patients (GEFSP) (N = 6), afebrile status epilepticus (SE) attacks in intractable epilepsy patients (N = 12), febrile controls without seizures (N = 41), First febrile seizure attack (FS) patients (N = 28) and recurrent FS attack patients (N = 13). (A) IL-1β levels are significantly high in both groups of afebrile SE and recurrent FS. (B) IL-6 levels are significantly high in the groups of first FS and recurrent FS (all, *p *< 0.05). (C and D) The trends toward high serum levels of TNF-α and HMGB1 in afebrile SE patients and recurrent FS patients, were statistically not significant (*p *= 0.06 and *p *= 0.11, respectively). Error bar, standard error of mean.

The mean IL-1β level of the afebrile control children was 2.0 pg/mL, while that for the febrile control was 3.1 pg/mL. The mean IL-1β level in afebrile status epilepticus attacks in intractable epilepsy patients was at a 11.7-fold increase higher than that of the afebrile controls (23.4 vs. 2.0 pg/mL) and a 7.5-fold increase higher than of fever only controls (Table 3, 23.4 vs. 3.1 pg/mL, *p *< 0.05). Comparisons of IL-1β levels among afebrile and febrile controls and the four seizure groups (first and recurrent febrile seizures, afebrile seizures in GEFSP and afebrile status epilepticus in intractable epilepsy patients) showed significantly higher levels in the afebrile status epilepticus in intractable epilepsy and the recurrent febrile seizure groups (Figure 1A, *p *< 0.05).

#### 2. IL-6

The mean IL-6 level of afebrile controls was 34.7 pg/mL, while that of febrile controls was 134.0 pg/mL, and that of afebrile status epilepticus attacks in intractable epilepsy patients was 51.4 pg/mL. Comparisons of IL-6 levels among afebrile and febrile controls, and the four seizure groups showed significantly higher IL-6 levels in first and recurrent attack febrile seizure patients (Figure 1B, p < 0.05).

#### 3. TNF-α

The mean TNF-α level of afebrile controls was 3.4 pg/mL, while that in febrile controls was 5.6 pg/mL, and that in afebrile status epilepticus attacks in intractable epilepsy patients was 14.2 pg/mL. Afebrile seizure patients showed a 57% decrease of TNF-α levels of febrile controls (Table 3, *p *< 0.05). Comparisons of TNF-α levels among afebrile and febrile controls, and the four seizure groups showed higher levels in the afebrile status epilepticus attacks in intractable epilepsy patients and the recurrent attack febrile seizure groups (Figure 1C, p = 0.06).

#### 4. HMGB1

The mean HMGB1 level in the serum of afebrile controls was 9.0 ng/mL, that in febrile control was 24.8 ng/mL, and that in afebrile status epilepticus attacks in intractable epilepsy patients was 30.1 ng/mL. In comparisons of HMGB1 levels between afebrile controls, febrile controls and the four seizure groups, there were trends of higher HMGB1 levels in both febrile seizures and afebrile status epilepticus attacks in intractable epilepsy patients than in the febrile and afebrile controls, but this was not statistically significant (Figure 1D, p = 0.11).

#### 5. IFN-γ

The mean IFN-γ level of afebrile controls was 20.8 pg/mL, that in febrile controls was 84.2 pg/mL, and that in afebrile status epilepticus attacks in intractable epilepsy patients was 21.4 pg/mL. Comparisons of IFN-γ levels among afebrile and febrile controls, and the four seizure groups showed no significant differences (Table 3).

#### 6. IL-10

The mean IL-10 level of afebrile controls was 2.7 pg/mL, that in febrile controls was 8.3 pg/mL, and that in afebrile status epilepticus attacks in intractable epilepsy patients was 0.6 pg/mL. Afebrile seizure patients and afebrile status epilepticus patients with intractable epilepsy showed significantly decreased IL-10 levels than that of febrile and afebrile controls (2.6 pg/mL & 0.6 pg/mL, *p *< 0.05). There were no significant differences of IL-10 levels among afebrile controls, febrile controls and the four seizure groups (Table 3).

### The correlations between the various cytokines

**Figure 2:**
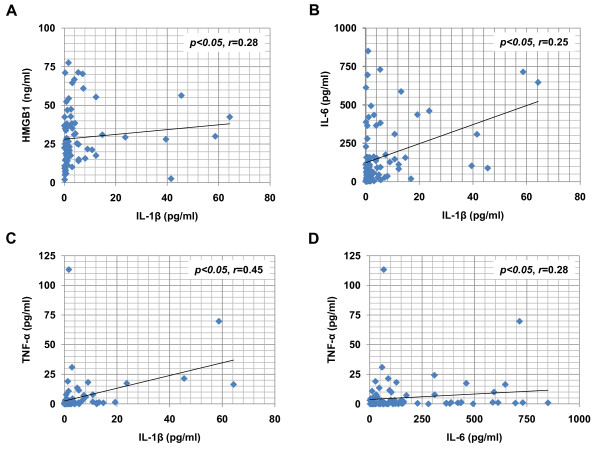
**Correlation between serum cytokine levels in seizure patients**. (A-D) Correlation between serum levels of IL-1β and HMGB1 (A), IL-1β and IL-6 (B), IL-1β and TNF-α (C), and IL-6 and TNF-α (D) in febrile patients (N = 82). IL-1β levels are significantly correlated with HMGB1, IL-6, and TNF-α levels (all, *p *< 0.05, *r *= 0.28, 0.25, and 0.45, respectively). IL-6 levels are significantly correlated with TNF-α levels (*p *< 0.05, *r *= 0.28).

IL-1β serum levels were significantly correlated with HMGB1, IL-6, and TNF-α levels (respectively: Figures 2A, B, and 2C; *r *= 0.28, *r *= 0.25, and *r *= 0.45; all *p *< 0.05), but not with IL-10 and IFN-γ. Serum IL-6 levels were significantly correlated with IL-1β and TNF-α levels (respectively: Figures 2B and 2D; *r *= 0.25 and *r *= 0.28; both *p *< 0.05), but were not correlated with HMGB1, IL-10, and IFN-γ levels.

## Discussion

This is the first study demonstrating a significant elevation of HMGB1 in the serum of febrile seizure patients. Moreover, serum levels of other pro-inflammatory cytokines, including IL-1β, IL-6, and the anti-inflammatory cytokine IL-10 were significantly higher among our patients with febrile seizures. IL-1β level increase was related to seizure recurrence and duration, as seen with the higher levels of IL-1β in recurrent febrile seizure or afebrile status epilepticus patients. In addition, IL-1β levels were significantly and positively correlated with HMGB1 levels and with other pro-inflammatory cytokines (IL-6 and TNF-α), supporting the association of the cytokine network in febrile seizures.

HMGB1 is a highly conserved, ubiquitously expressed protein [18] and is actively secreted from monocytes and macrophages in response to challenges with LPS [19]. HMGB1 binds to and transfers LPS, consequently increasing LPS-induced TNF-α production in human peripheral blood mononuclear cells [13]. HMGB1 is passively released from necrotic cells, but not from apoptotic cells, thereby creating a signal for the organism to distinguish between the two types of cell death [20]. Several clinical studies have reported that serum HMGB1 levels are elevated in patients with infection and/or systemic inflammatory response syndrome, than in healthy control individuals [19,21]. HMGB1 is involved in various diseases without obvious infections; for example, rheumatoid arthritis [22], hemorrhagic shock [23], cerebral and myocardial ischemia [24], acute lung injury [25], and acute pancreatitis [26]. HMGB1 is highly expressed in human epileptogenic brain, and antagonists of HMGB1 and TLR4 have been demonstrated to retard seizure precipitation and to decrease acute and chronic seizure recurrence in epilepsy animals [15]. These findings suggest a role for the HMGB1-TLR4 axis in epilepsy. In our study, serum levels of HMGB1 were significantly higher in febrile seizure patients and showed a positive correlation with IL-1β levels. Our results, together with those from other studies, suggest that HMGB1 activation is an important feature associated with epilepsy and febrile seizures.

IL-1β serum levels were significantly higher in our febrile seizures patients than in febrile children without seizures. IL-1β has been shown to have potent pro-convulsant properties in experimental animals [27]. IL-1β acts on astrocytes to increase glutamate release via TNF-α production [28], resulting in elevated extracellular glutamate levels and hyper-excitability. Also, IL-1β can stimulate IL-6 release [29]. In our patients, IL-1β levels were significantly correlated with IL-6, HMGB1, and TNF-α levels. In our previous work using epileptogenic brain cortices of children with intractable epilepsy, pro-inflammatory cytokines, IL-1β, IL-8, IL-12p70, and MIP-1β were increased significantly above those in non-epileptogenic control brain cortices [30]. Our patients with intractable epilepsy experiencing status epilepticus attacks also showed high IL-1β, IL-6 and HMGB1 levels. These results together suggest that active inflammation does occur in febrile seizures and pediatric epilepsy, and it may play a common pathologic role in febrile seizures and epilepsy.

Since cytokine levels were measured with blood taken 30 min after the seizure, the acute effect of seizures could not be distinguished from a persistent inflammatory tone in febrile seizure patients. Seizures themselves can activate the sympathetic nervous system and induce the release of catecholamines [31,32], resulting in cytokine release from peripheral blood mononuclear cells [33]. However, in our study, patients with recurrent febrile seizure attacks had much higher IL-1β and TNF-α levels than patients with first attack febrile seizures, although interictal cytokine levels were not available after acute seizures. In animal models of prolonged febrile seizures, IL-1β was significantly high in the hippocampus for over 24 hours and was elevated chronically only in rats developing spontaneous limbic seizures after febrile status epilepticus [9,34]. These findings suggest that inflammatory responses in febrile seizures are accentuated by their repetition and increase the likelihood of febrile seizure recurrence.

Interestingly, no increase in serum IL-1β was detected in our children with fever but no seizures (3.1 pg/mL), as compared to controls without fever and with no seizures (2.0 pg/mL). In another study, similarly low IL-1β blood levels of 3.4 pg/mL were reported in the febrile control group [35]. This lack of increase may be the result of excluding patients with presumptive bacterial infections, because LPS is the main inducer for the synthesis of IL-1β [36]. Also, IL-1β is usually difficult to detect because of its binding to large proteins such as α-2 macroglobulin and complement [37]. Furthermore, fever could occur independently of IL-1 or TNF activity during infections, and the cytokine-like property of TLR signal transduction could be one explanation [38].

The sources of the serum cytokine in febrile seizures patients are not clear. The main source of IL-1 is monocytes in the periphery and microglial cells in the nervous system, which upon activation secrete the cytokines. Cytokines are produced by astrocytes and some neurons in the CNS by LPS and other stimuli [39,40]. Under normal conditions, the levels of IL-1 are low, both in the circulation and in the CNS, whereas upon infection or injury, IL-1 levels increase abruptly but transiently, returning to normal within 8 h in healthy, young mouse brain [41]. Therefore, high serum levels of IL-1β may reflect high levels in the CNS. However, conflicting results about IL-1β levels have been reported in peripheral blood and CSF of children with febrile seizures, such as high in plasma but not in CSF [42], or high in CSF but not in serum [43] or increase in neither serum nor CSF [35]. These results potentially reflect difficulties in obtaining clinical samples and measuring free IL-1β. The possible sources of the serum cytokine increases in febrile seizures may be peripheral mononuclear cells, CSF-blood exchange, and leakage from the brain reticuloendothelial system.

A dual role of IL-6 in seizures has been demonstrated in several animal models. IL-6 knockout mice showed an increased seizure susceptibility to glutamate receptor agonists [44]. Transgenic mice over-expressing IL-6 in astrocytes were also reported to have an increased seizure susceptibility to glutamate receptor agonists, probably due to reduced GABA-mediated inhibition [45]. In developing rats, intra-nasal administration of IL-6 prolonged the latency and shortened the duration of hyperthermia-induced seizures, suggesting an anti-convulsant effect to febrile seizures [46]. On the other hand, intranasal administration of IL-6 in adult rats exacerbated the severity of seizures induced by pentylenetetrazole, supporting a pro-convulsant effect [47]. In our patients including only presumptive viral infections, serum IL-6 was higher in febrile seizure children than in fever only controls. Moreover, IL-6 levels in febrile seizure patients were much higher than in afebrile seizure attack patients. Higher IL-6 and IFN-α levels have been reported in patients with influenza-associated febrile seizures compared to those without febrile seizures [3,48]. These findings, with our results, may support the pro-convulsant action of IL-6 in febrile seizures.

IL-10 is a multifunctional anti-inflammatory cytokine produced by monocytes, macrophages, lymphocytes, as well as microglia and inhibits the production of pro-inflammatory cytokines, including IL-1, IL-6, IL-8, and TNF-α [49]. Peripheral blood mononuclear cells from febrile seizure patients have shown increased IL-10 production by LPS [50]. In IL-10 injected animals, the febrile seizure threshold was significantly higher than that in controls, suggesting that IL-10 is associated with a resistance to febrile seizures [51]. Previously, plasma IL-10 levels showed no difference between febrile seizures and controls [3]. However, in our patients, IL-10 levels were higher in recurrent febrile seizure patients than in first attack febrile seizure patients and were also higher in patients without a family history of febrile seizures than in patients with family history. These findings may reflect compensatory activation of anti-inflammatory or anti-convulsive mechanisms, or mechanism defects in the anti-inflammatory role of IL-10 in febrile seizure families; further studies into the role of IL-10 are warranted.

TNF-α causes both detrimental and beneficial effects on brain function depending on its concentration, targeted cells, duration of exposure and the specific receptor subtypes [52,53]. TNF-α is rapidly upregulated in the CNS by seizures, and intrahippocampal injection of TNF-α potently inhibit seizure in a mice model of epilepsy [54]. In our children with acute and brief seizures, either febrile or afebrile, serum TNF-α was decreased, or at least not increased, supporting that TNF-α is not involved in the mechanisms by which seizures are triggered. On the other hand, transgenic mice over-expressing high amounts of TNF-α in astrocytes developed spontaneous seizures, [55] and TNF-α has been shown to increase excitatory postsynaptic currents in hippocampal neurons [56] and to decrease GABA_A_-mediated inhibitory synaptic strength, leading to increased seizure susceptibility [57,58]. Our recurrent febrile seizure patients showed higher serum TNF-α levels than first attack febrile seizure patients, and afebrile status epilepticus attacks in intractable epilepsy patients showed higher serum TNF-α level than short-duration seizure attacks in GEFSP patients, supporting that chronic or recurrent expression of TNF-α may change susceptibility to seizures.

The causative role of cytokines in epileptogenesis remains to be elucidated. Cytokines may contribute initially to incite seizures in the developing brain after being induced by seizure or tissue injury, and they may exacerbate tissue injury and promote further seizures. Furthermore, cytokine gene polymorphisms have been linked to epilepsy susceptibility [4]. Thus, it may be worthwhile to explore further a possible link between febrile seizures and genetic susceptibility to inflammation.

In summary, HMGB1 and pro-inflammatory cytokines were significantly higher in febrile seizure patients. Although it is not possible to infer causality from descriptive human studies, our data suggest that HMGB1 and the cytokine network may contribute to the generation of febrile seizures in children. Pro-inflammatory cytokine production may promote seizures, further exacerbate epilepsy, and may cause subsequent intractable epilepsy. If so, there may be a potential role for anti-inflammatory therapy targeting cytokines and HMGB1 as a novel therapeutic strategy to prevent or limit febrile seizures or subsequent epileptogenesis in the vulnerable, developing nervous system of children.

## Competing interests

The authors declare that they have no competing interests.

## Authors' contributions

JC reviewed and helped in analyzing data, obtained IRB approval and permissions from the patients and their parents, processed serum from the patients, conducted cytokine analyses, and helped draft and prepare the manuscript for publication. HM performed the HMGB1 ELISA analyses. JS reviewed and helped in the data analyses as well as helped with drafting and preparing the manuscript for publication. All authors have read and approved the final version of the manuscript.
